# A computational biomarker of idiopathic generalized epilepsy from resting state EEG

**DOI:** 10.1111/epi.13481

**Published:** 2016-08-08

**Authors:** Helmut Schmidt, Wessel Woldman, Marc Goodfellow, Fahmida A. Chowdhury, Michalis Koutroumanidis, Sharon Jewell, Mark P. Richardson, John R. Terry

**Affiliations:** ^1^ College of Engineering, Mathematics & Physical SciencesUniversity of ExeterExeterUnited Kingdom; ^2^ Wellcome Trust ISSF Centre for Biomedical Modelling and AnalysisUniversity of ExeterExeterUnited Kingdom; ^3^ EPSRC Centre for Predictive Modelling in HealthcareUniversity of ExeterExeterUnited Kingdom; ^4^ Institute of Psychiatry, Psychology & NeuroscienceKing's College LondonLondonUnited Kingdom; ^5^ Department of EEG and EpilepsySt. Thomas's HospitalGuy's and St. Thomas's NHS Foundation TrustLondonUnited Kingdom

**Keywords:** Biomarker, Diagnosis, Resting‐state EEG, Computational model, IGE

## Abstract

Epilepsy is one of the most common serious neurologic conditions. It is characterized by the tendency to have recurrent seizures, which arise against a backdrop of apparently normal brain activity. At present, clinical diagnosis relies on the following: (1) case history, which can be unreliable; (2) observation of transient abnormal activity during electroencephalography (EEG), which may not be present during clinical evaluation; and (3) if diagnostic uncertainty occurs, undertaking prolonged monitoring in an attempt to observe EEG abnormalities, which is costly. Herein, we describe the discovery and validation of an epilepsy biomarker based on computational analysis of a short segment of resting‐state (interictal) EEG. Our method utilizes a computer model of dynamic networks, where the network is inferred from the extent of synchrony between EEG channels (functional networks) and the normalized power spectrum of the clinical data. We optimize model parameters using a leave‐one‐out classification on a dataset comprising 30 people with idiopathic generalized epilepsy (IGE) and 38 normal controls. Applying this scheme to all 68 subjects we find 100% specificity at 56.7% sensitivity, and 100% sensitivity at 65.8% specificity. We believe this biomarker could readily provide additional support to the diagnostic process.

At present, a confirmed diagnosis of epilepsy is made through a case history and a positive electroencephalography (EEG), confirming the presence of epileptiform discharges. However, a positive EEG occurs at best in only 60% of cases, resulting in diagnostic uncertainty for many people,^1^ with significant associated costs.^2^ These costs predominantly result from additional longer‐term EEG monitoring, repeated hospital admissions, as well as unnecessary prescription of antiepileptic drugs (AEDs).

Idiopathic generalized epilepsy (IGE) is one of the main classes of epilepsy. In recent years, studies comparing cohorts of people with IGE and cohorts of healthy controls have shown statistically significant alterations at the group level when examining resting‐state features of the EEG using power spectrum,^3^ functional networks,^4^ and a model‐driven analysis of functional networks.^5^ However, substantial overlap of these markers between groups may render the measurement unsuitable as a diagnostic test or biomarker^6^ for any one individual. Our aim therefore is to assess the performance of each of these methods as a classifier that has three outcomes for each individual: unequivocally IGE, unequivocally normal, or uncertain. Such a classifier could be used as a screening test in a nonspecialist primary care setting, as well as a diagnostic validation test in a specialist epilepsy setting. This would focus further medical investigation and resources on a smaller subgroup, producing efficiency gains and cost savings.

## Methods

We studied data from 38 healthy controls and 30 people with IGE between the ages of 16 and 59 years. The individuals with IGE were drug naive and recruited through clinics at St Thomas's Hospital. A diagnosis of epilepsy was confirmed in each case by an experienced epilepsy specialist through observation of typical generalized spike‐wave (GSW) activity on EEG either spontaneously or following hyperventilation or photic stimulation. For 10 of these people, the diagnosis was confirmed following an initial routine EEG. For the remaining 20, diagnosis was confirmed following sleep‐deprived or longer‐term EEG monitoring (including sleep). Similar healthy control EEG was collected at King's College Hospital EEG department. Controls provided written informed consent, and data collection was approved by King's College Hospital Research Ethics Committee (08/H0808/157). Under United Kingdom law, patient data collected during normal clinical routine and anonymized before research use may be used for research without additional consent; this procedure was reviewed and approved for this project by St. Thomas's Hospital and King's College Hospital's Research and Development departments.

A trained clinical EEG technician identified a 20‐s‐long GSW and artifact‐free segment of eyes‐closed “resting state” EEG activity from the initial stage of the recordings from each participant. These data were band‐pass filtered using a Butterworth filter between 0.5 and 70 Hz, and band‐stop filtered between 48 and 52 Hz to remove power‐line artifacts. Because signal amplitude may vary between individuals due to different anatomic features (such as the size and shape of the cranium) the data were normalized by dividing the power spectrum in each channel by the total power in the spectrum averaged across all channels. This normalized power preserves relative differences in power between channels. We then band‐pass filtered the EEG segments into either the alpha (8–13 Hz) or low alpha bands^7^ (6–9 Hz). For segments band‐pass filtered in the low alpha band, we further inferred functional networks using the Phase‐Locking Factor^8^ (PLF) and phase‐lags (as described previously).^5^

**Figure 1:**
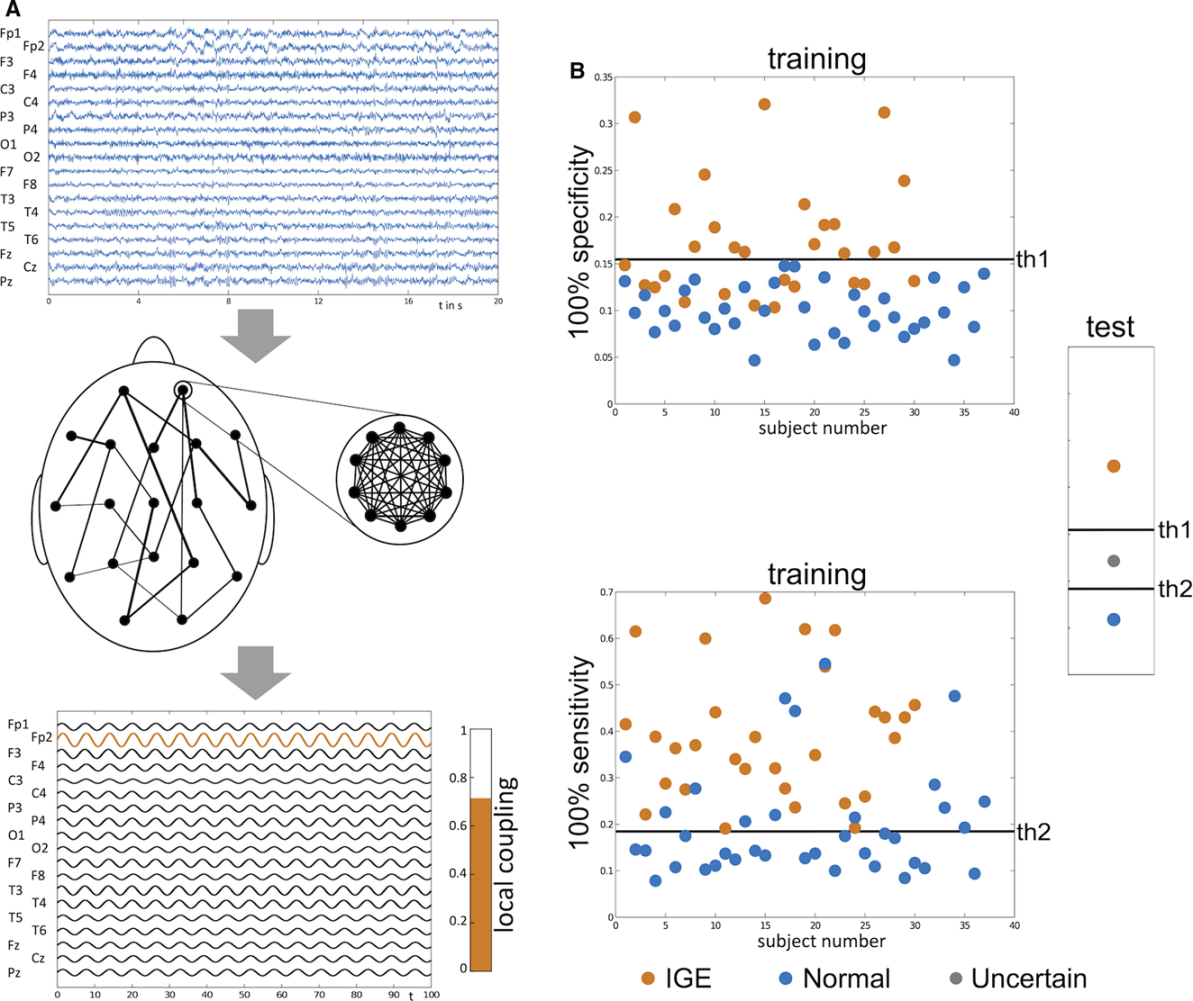
Schematic of acquiring the local coupling biomarker and illustrative performance assessment. (**A**) The local coupling biomarker, which was identified as the best performing biomarker in this study, is acquired by inferring the global (between‐channel) network structure and the local (within‐channel) coupling strength from resting‐state EEG (top panel), and incorporating them into an oscillator model. In this scenario, each node within the network corresponds to an EEG channel (middle panel). The biomarker is quantified by placing each node within the model into a state of synchrony (by increasing its internal coupling strength beyond a threshold), and the level of emergent synchrony across the whole network is calculated (bottom panel). This level of synchrony across the network is the model proxy for seizures, which might be thought of as a “seizure likelihood.” This biomarker depends on channel location and model parameters, and thus we can perform procedures to optimize its performance. (**B**) To assess the performance of this biomarker as a classifier, we use the leave‐one‐out approach, in which one subject is left aside, and test subject and all other subjects form the training set on which parameters are optimized. This optimization process results in a threshold for the biomarker (th1) that yields the highest level of sensitivity at 100% specificity and another optimized set of parameters and threshold (th2) that yields the highest level of specificity at 100% sensitivity. This is illustrated for a single realization of the leave‐one‐out approach in the top and bottom panels. These thresholds are then applied to the test subject, where the outcome will be “IGE,” “normal,” or “uncertain” depending on where the value of the biomarker for the test subject lies.

For the purpose of biomarker discovery, we consider measures that have demonstrated group‐level differences between people with IGE and healthy controls using resting‐state EEG. First, the peak in alpha power across occipital EEG channels, which is known to shift toward lower frequencies in people with IGE.^3^ Second, the mean degree of the PLF‐inferred low alpha functional network, which is elevated in people with IGE.^4^ Third, a model‐driven analysis where the low alpha functional network inferred from the EEG of each individual is integrated within a phase oscillator model (of Kuramoto type).^5^ Here the local coupling constant within each node of the network is inferred by multiplying the variance of the signal in the corresponding EEG channel by a uniform parameter K, to give a subject‐specific dynamic network model of the brain. The seizure‐generating capability of each region within this model is then evaluated computationally, as the average level of emergent seizure activity across the whole network driven by the region of interest (see Fig. 1A).

The performance of all three candidate biomarkers was evaluated using “leave‐one‐out” classification,^9^ in which all 30 people with IGE and 38 controls are pooled, the data from one subject is successively left aside, and the remaining data is used as the training set. In each case, thresholds are determined to give the highest sensitivity for 100% specificity and the highest specificity for 100% sensitivity in the training set. In turn, these thresholds are applied to classify the test subject as follows: If the value of local coupling is on the IGE side of both thresholds, then the individual is classified as unequivocally having epilepsy. The individual is classified as unequivocally normal if their value is on the control side of both thresholds. If their value lies between these thresholds they are classified as uncertain. A graphical representation of this approach is shown in Figure 1B. Because each outcome is discrete and non‐normal, we use the Friedman test^10^ (nonparametric repeated measures analysis of variance [ANOVA]) to assess the relative performance of each biomarker.

## Results

Successively optimizing the channel location and value of the local coupling constant to give the highest levels of sensitivity and specificity in each training set, the local coupling biomarker resulted in 56.7% sensitivity (given 100% specificity) and 65.8% specificity (given 100% sensitivity). Specifically, 17 of 30 people with IGE were classified as unequivocally having epilepsy, 10 received an uncertain classification, and 3 were misclassified. Of the 38 healthy controls, 25 were correctly classified and 13 received an uncertain classification.

In contrast, average power of the EEG power spectrum and the mean degree of the inferred functional network performed poorly with low sensitivity and specificity. The peak in alpha power resulted in 0% sensitivity (given 100% specificity) and 0% specificity (given 100% sensitivity). It classified no people with IGE as having epilepsy; 29 were classified as uncertain and one was misclassified. Of the 38 healthy controls, none were correctly classified, 37 received an uncertain classification, and one was misclassified. Mean degree resulted in 3.3% sensitivity (given 100% specificity) and 15.8% specificity (given 100% sensitivity). It classified one person with IGE as having epilepsy, 28 were classified as uncertain, and one was misclassified. Of the 38 healthy controls, 6 were correctly classified, 31 received an uncertain classification, and one was misclassified.

The Friedman test confirms that the classification results of the local coupling biomarker are statistically significant for people with IGE (χ^2^ = 26.77, p < 0.001) and controls (χ^2^ = 22.83, p < 0.001) in comparison to the other potential biomarkers. Using pairwise comparison, we show that the local coupling performs consistently better than either average power (IGE: χ^2^ = 14.22, p < 0.001; controls: χ^2^ = 7.14, p = 0.007) or mean degree (IGE: χ^2^ = 13.24, p < 0.001; controls: χ^2^ = 19.17, p < 0.001).

## Discussion

Herein we describe the comparative analysis of three candidate biomarkers of IGE using 20 s segments of “resting‐state” EEG from cohorts of drug naive people with IGE and age‐ and gender‐matched healthy controls. To our knowledge these three candidates are the only published methods to date that have shown statistically significant differences at the group level using “resting‐state” EEG. The best performing algorithm, based upon a computer model of local and global brain networks, achieved nearly 60% sensitivity given 100% specificity and >60% specificity given 100% sensitivity. We assessed performance in this manner, since an ideal screening test to use in a nonspecialist setting needs 100% sensitivity to ensure all people with IGE are captured (but some false positives are tolerable), whereas a decision support tool in the specialist setting needs 100% specificity to avoid false positives, but less than perfect sensitivity can be compensated for by further expert‐driven evaluation.

The use of routinely acquired EEG data, combined with minimal computational cost for evaluating the biomarker, makes this an attractive proposition from the perspective of clinical decision support. At present, the most time‐consuming part is visual identification of “resting‐state” EEG, which in our study was performed by a trained EEG technician. Automating this process would permit delivery of a result in real‐time (potentially while EEG was still being collected). A critical advantage of this method is that there is no requirement to observe epileptiform discharges in EEG to make a diagnosis, since the method relies only on brief segments of “resting‐state” EEG. This yields the potential for a screening service to be offered in a nonspecialist primary care environment, a resource‐poor setting, or even using nonspecialist EEG carried out in the patient's home.

Although these results are promising, it is important to note potential confounds that may limit the sensitivity and specificity achievable. Of note, cortical excitability (and by assumption seizure likelihood) is known to vary according to time of day; varying in response to both physiologic factors and external stimuli.^11^ It has very recently been shown that endocrine activity displays the strongest relationship with this circadian change.^12^ In this study, most recordings were taken in the late morning or early afternoon and we found no significant difference (Wilcoxon test: p = 1, *t*‐test: p = 0.758) in the times when recordings were taken and whether a subject was correctly classified (mean time – 12:52 ± 1:36) or not (mean time – 12:38 ± 2:19).

The full code written in MATLAB^13^ can be found online (Data S1).

## Disclosure of Conflict of Interest

Helmut Schmidt, Mark P. Richardson, and John R. Terry received financial support from Epilepsy Research UK (via Grant A1002). Marc Goodfellow, Mark P. Richardson, and John R. Terry received financial support from the Medical Research Council (via Programme Grant MR/K013998/1) and the EPSRC (via Centre Grant EP/N014391/1). John R. Terry further acknowledges the generous support of the Wellcome Trust Institutional Strategic Support Award (WT105618MA). Mark P. Richardson is part‐funded by the National Institute of Health Research (NIHR) Biomedical Research Centre at the South London and Maudsley NHS Foundation Trust. The remaining authors have no conflict of interest to declare. We confirm that we have read the Journal's position on issues involved in ethical publication and affirm that this report is consistent with those guidelines.

## Supporting information


**Data S1.** MATLAB scripts.
