# Supplementary material for: A computational biomarker of idiopathic generalized epilepsy from resting state EEG
Source: Epilepsia. 2016 Aug 8;57(10):e200–4. doi: 10.1111/epi.13481 (PMC5082517; doi:10.1111/epi.13481)
Supplement: Supplementary file 1 — Data S1. MATLAB scripts. [file EPI-57-e200-s001.pdf]

```
%%%%%%%%%%%%%%%%%%%%%%%%%%%%%%%%%%%%%%%%%%%%%%%%%%%%%%%%%%%%%%%%%%%%%%%%%%%%
% OUTLINE
%%%%%%%%%%%%%%%%%%%%%%%%%%%%%%%%%%%%%%%%%%%%%%%%%%%%%%%%%%%%%%%%%%%%%%%%%%%
% Main scripts:
%     sComputeMeasures
%         Computes the three candidate biomarkers after loading EEG (to be
%         placed in folders 'Control' and 'IGE'). Computed values are stored
%         in 'results.mat'. Currently, the recordings are expected to be in
%         mat-format; in case of edf-files, our advice is to use
%         lab_read_edf() (see file exchange)
%
%   sClassifyLOO
%         Runs leave-one-out cross validation for values computed by
%         sComputeMeasures (stored in 'results.mat). Classifies all
%         individuals using leave-one-out cross-validation, and computes
%         sensitivity and specificity for the three candidate biomarkers
%
% Auxiliary functions:
%   fAlphaPeak
%         Computes peak in alpha-band (Larson et al. 2005)
%   fBandPass
%         Standard bandpass filter for a time series
%     fBiomarker
%         Computes the three candidate biomarkers
%   fDespur
%         Method to remove spurious connections (Schmidt et al. 2014)
%   fLocal
%         Compute Kuramoto-outcome for local coupling (Schmidt et al. 2014)
%   fSEN
%         Computes maximum sensitivity and threshold for 100% specificity
%   fSPC
%         Computes maximum specificity and threshold for 100% sensitivity
%   fSpectrum
%         Compute frequency-spectrum for a time series
%
% Other:
%   results.mat
%         Generated by running ComputeMeasures.m; contains 2 cells: IGE &
%         CON, containing the computed values for the three candidate
%         biomarkers (Alpha, MeanDeg, Local)

%%%%%%%%%%%%%%%%%%%%%%%%%%%%%%%%%%%%%%%%%%%%%%%%%%%%%%%%%%%%%%%%%%%%%%%%%%%
% SCRIPT: sComputeMeasures.m
%%%%%%%%%%%%%%%%%%%%%%%%%%%%%%%%%%%%%%%%%%%%%%%%%%%%%%%%%%%%%%%%%%%%%%%%%%%
% Compute all biomarkers, for 30 subjects with IGE, and 38 controls.
% Precomputing speeds up parameter optimisation and thresholding for
% leave-one-out cross-validation. The data are given as follows:
%   Controls: in folder 'Control' with cell 'EEG' and elements 'data'
%   containing the recorded time series, and 'dt' the time between
%   two samples
%   IGE: in folder 'IGE' with cell 'EEG' and elements 'data'
%   containing the recorded time series, and 'dt' the time between
%   two samples

% Clear variables and initialize display function
warning('off'); close all; clear all;
nCON = 38;
nIGE = 30;
```

```
% Compute biomarkers for control subjects
for k = 1:nCON
    filename = strcat('Control/Control',int2str(k),'.mat');
    load(filename,'-mat');
    eegdata = EEG.data;
    dt = 1./EEG.srate;
    [CON{k}.Alpha,CON{k}.MeanDeg,CON{k}.Local] = fBiomarker(eegdata,dt);
end

% Compute biomarkers for IGE subjects
for k = 1:nIGE
    filename = strcat('IGE/IGE',int2str(k),'.mat');
    load(filename,'-mat');
    eegdata = EEG.data;
    dt = 1./EEG.srate;
    [IGE{k}.Alpha,IGE{k}.MeanDeg,IGE{k}.Local] = fBiomarker(eegdata,dt);
end

save('Results.mat','CON','IGE');
% END OF FILE



%%%%%%%%%%%%%%%%%%%%%%%%%%%%%%%%%%%%%%%%%%%%%%%%%%%%%%%%%%%%%%%%%%%%%%%%%%%%%
% SCRIPT: sClassifyLOO.m
%%%%%%%%%%%%%%%%%%%%%%%%%%%%%%%%%%%%%%%%%%%%%%%%%%%%%%%%%%%%%%%%%%%%%%%%%%%%%
% This script performs the leave-one-out classification on biomarkers
% computed with script 'sComputeMeasures.m', outcome saved in 'Results.mat'
%
% Leave-one-out cross validation sets aside one subject as test set, and
% uses all other subjects as training set. For the training set we
% determine two thresholds: one at which the classifier has 100%
% sensitivity (at maximum specificity), and one at which the classifier has
% 100% specificity (at maximum sensitivity). These two thresholds are then
% applied to the test subject. If the test subject is above or below both
% thresholds, it is then classified as either normal or with epilepsy. If
% it falls between the thresholds, it is classified as uncertain. This
% process is repeated until all subjects have been tested exactl one, after
% which sensitivity and specificity are reported.

load('Results.mat')
nCon = 38;
nIGE = 30;
nCH = 19;
% preallocate vectors for loading computed measures (from results.mat)
ConAlpha=zeros(nCon,1);ConMeanDeg=zeros(nCon,1);ConLocal=zeros(nCon,19,20);
IGEAlpha=zeros(nIGE,1);IGEMeanDeg=zeros(nIGE,1);IGELocal=zeros(nIGE,19,20);
for m = 1:nCon
    ConAlpha(m,1) = Control{m}.Alpha;
    ConMeanDeg(m,1) = Control{m}.MeanDeg;
    ConLocal(m,:,:) = abs(Control{m}.Local);
end
for m = 1:nIGE
    IGEAlpha(m,1) = IGE{m}.Alpha;
    IGEMeanDeg(m,1) = IGE{m}.MeanDeg;
    IGELocal(m,:,:) = IGE{m}.Local;
end
```

```
%% Test Alpha Peak Biomarker:
ThSenA = zeros(1,nCon+nIGE);
ThSpcA = zeros(1,nCon+nIGE);
for k = 1:nCon+nIGE
    % leave k-th subject out:
    if k<=30
        pID = setdiff(1:nIGE,k);
        cID = 1:nCon;
        ThisA = IGEAlpha(k);
    else
        pID = 1:nIGE;
        cID = setdiff(1:nCon,k-nIGE);
        ThisA = ConAlpha(k-nIGE);
    end
    % find thresholds for sensitivity and specificity:
    [~,thrSE] = fSEN(IGEAlpha(pID),ConAlpha(cID));
    [~,thrSP] = fSPC(IGEAlpha(pID),ConAlpha(cID));
    % check whether subject is above or below thresholds:
    ThSenA(k) = 1.*(ThisA<thrSE);
    ThSpcA(k) = 1.*(ThisA<thrSP);
end
% Classify subjects:
% if both equal 1, than classified as IGE
ClassAsIGEAlpha = 1.*(ThSenA&ThSpcA);
% if both equal 0, then classifed as CON
ClassAsConAlpha = 1.*(~(ThSenA|ThSpcA));
% if a 0 and a 1, then UNCERTAIN
ClassAsUnAlpha = 1.*xor(ThSenA,ThSpcA);

%% Test Mean-Degree Biomarker:
ThSenMD = zeros(1,nCon+nIGE);
ThSpcMD = zeros(1,nCon+nIGE);
for k = 1:nCon+nIGE
    % leave k-th subject out:
    if k<=nIGE
        pID = setdiff(1:nIGE,k);
        cID = 1:nCon;
        ThisMD = IGEMeanDeg(k);
    else
        pID = 1:nIGE;
        cID = setdiff(1:nCon,k-nIGE);
        ThisMD = ConMeanDeg(k-nIGE);
    end
    % find thresholds for sensitivity and specificity:
    [~,thrSE] = fSEN(IGEMeanDeg(pID),ConMeanDeg(cID));
    [~,thrSP] = fSPC(IGEMeanDeg(pID),ConMeanDeg(cID));
    % check whether subject is above threshold or not:
    ThSenMD(k) = 1.*(ThisMD>thrSE);
    ThSpcMD(k) = 1.*(ThisMD>thrSP);
end
% Classify subjects:
ClassAsIGEMeanDeg = 1.*(ThSenMD&ThSpcMD);
ClassAsConMeanDeg = 1.*(~(ThSenMD|ThSpcMD));
ClassAsUnMeanDeg = 1.*xor(ThSenMD,ThSpcMD);

%% Test Local Coupling Biomarker:
%ThSenL = zeros(1,nCon+nIGE);
%ThSpcL = zeros(1,nCon+nIGE);
```

```
for k = 1:nCon+nIGE
    % leave k-th subject out:
    if k<=nIGE
        pID = setdiff(1:nIGE,k);
        cID = 1:nCon;
        ThisL = squeeze(IGELocal(k,:,:));
    else
        pID = 1:nIGE;
        cID = setdiff(1:nCon,k-nIGE);
        ThisL = squeeze(ConLocal(k-nIGE,:,:));
    end
    % find thresholds for sensitivity and specificity across parameters:
    for c = 1:nCH
        for r = 1:20
            [SE(c,r),thrSE(c,r)]=fSEN(IGELocal(pID,c,r),ConLocal(cID,c,r));
            [SP(c,r),thrSP(c,r)]=fSPC(IGELocal(pID,c,r),ConLocal(cID,c,r));
        end
    end
    % reshape threshold arrays:
    thrSE2 = thrSE(:);
    thrSP2 = thrSP(:);
    % find parameter combinations with highest sensitivity and specificity:
    [~,senID] = max(SE(:));
    [~,spcID] = max(SP(:));
    % reshape array ThisL:
    ThisL2 = ThisL(:);
    % check whether subject is above or below thresholds:
    ThSenL(k) = 1.*(ThisL2(senID)>thrSE2(senID));
    ThSpcL(k) = 1.*(ThisL2(spcID)>thrSP2(spcID));
end
% Classify subjects:
ClassAsIGELocal = 1.*(ThSenL&ThSpcL);
ClassAsConLocal = 1.*(~(ThSenL|ThSpcL));
ClassAsUnLocal = 1.*xor(ThSenL,ThSpcL);

%% Results
% for Alpha-biomarker
% number of subjects with IGE correctly classified as IGE
AIGE_IGE=sum(ClassAsIGEAlpha(1:nIGE));
% number of subjects with IGE classified as uncertain
AIGE_Un=sum(ClassAsUnAlpha(1:nIGE));
% number of subjects with IGE classified as control
AIGE_Con=sum(ClassAsConAlpha(1:nIGE));
% number of controls classified as IGE
ACon_IGE=sum(ClassAsIGEAlpha(nIGE+1:nCon+nIGE));
% number of controls classified as uncertain
ACon_Un=sum(ClassAsUnAlpha(nIGE+1:nCon+nIGE));
% number of controls correctly classified as control
ACon_Con=sum(ClassAsConAlpha(nIGE+1:nCon+nIGE));
% for Mean Degree-biomarker
MIGE_IGE=sum(ClassAsIGEMeanDeg(1:nIGE));
MIGE_Un=sum(ClassAsUnMeanDeg(1:nIGE));
MIGE_Con=sum(ClassAsConMeanDeg(1:nIGE));
MCon_IGE=sum(ClassAsIGEMeanDeg(nIGE+1:nCon+nIGE));
MCon_Un=sum(ClassAsUnMeanDeg(nIGE+1:nCon+nIGE));
MCon_Con=sum(ClassAsConMeanDeg(nIGE+1:nCon+nIGE));
% for local coupling biomarker
LIGE_IGE=sum(ClassAsIGELocal(1:nIGE));
```

```
LIGE_Un=sum(ClassAsUnLocal(1:nIGE));
LIGE_Con=sum(ClassAsConLocal(1:nIGE));
LCon_IGE=sum(ClassAsIGELocal(nIGE+1:nCon+nIGE));
LCon_Un=sum(ClassAsUnLocal(nIGE+1:nCon+nIGE));
LCon_Con=sum(ClassAsConLocal(nIGE+1:nCon+nIGE));

%% Sensitivity and specificity
% for Alpha-biomarker:
AlphaSEN = (AIGE_IGE/nIGE)*100;
AlphaSPEC = (ACon_Con/nCon)*100;
MDegSEN = (MIGE_IGE/nIGE)*100;
MDegSPEC = (MCon_Con/nCon)*100;
LocalSEN = (LIGE_IGE/nIGE)*100;
LocalSPEC = (LCon_Con/nCon)*100;

%% Display results
fprintf(['Biomarker: Alpha\nIGE: %d IGE, %d Unc, %d Con.', ...
'\nControls: %d IGE, %d Unc, %d Con.', ...
'\nSensitivity: %.3g%%, Specificity: %.3g%%', ...
'\n\nBiomarker: Mean Degree\nIGE: %d IGE, %d Unc, %d Con.', ...
'\nControls: %d IGE, %d Unc, %d Con.', ...
'\nSensitivity: %.3g%%, Specificity: %.3g%%', ...
'\n\nBiomarker: Local\nIGE: %d IGE, %d Unc, %d Con.', ...
'\nControls: %d IGE, %d Unc, %d Con.', ...
'\nSensitivity: %.3g%%, Specificity: %.3g%%\n'], ...
AIGE_IGE,AIGE_Un,AIGE_Con,ACon_IGE,ACon_Un,ACon_Con,AlphaSEN,AlphaSPEC,...
MIGE_IGE,MIGE_Un,MIGE_Con,MCon_IGE,MCon_Un,MCon_Con,MDegSEN,MDegSPEC,...
LIGE_IGE,LIGE_Un,LIGE_Con,LCon_IGE,LCon_Un,LCon_Con,LocalSEN,LocalSPEC);
% END OF FILE



%%%%%%%%%%%%%%%%%%%%%%%%%%%%%%%%%%%%%%%%%%%%%%%%%%%%%%%%%%%%%%%%%%%%%%%%%%%%%
%% Auxiliary functions
function [Alpha,MeanDeg,Local] = fBiomarker(mydata,dt)
% This function computes the following candidate biomarkers:
%   Alpha   - alpha peak frequency
%   MeanDeg - mean degree of the functional network (PLF)
%   Local   - local coupling biomarker, which tests individual channel
%             locations for their ictogenicity
% AUXILIARY FUNCTIONS:
%   fBandpass   - bandpass filters data in given frequency band
%   fDespur     - computes sparse connectivity matrix
%   fAlphaPeak  - computes maximum alpha peak
%   fLocal      - computes local coupling for given set of parameters

% ensure first dimension of array is EEG channel:
if size(mydata,1)>size(mydata,2);
    mydata = mydata';
end
nCH = 19;
% define low-alpha band:
frq = [6;9];
% reference against average:
mydata = mydata - repmat(mean(mydata,1),[size(mydata,1) 1]);
% select 20s segment (shorter if not possible)
Nt = floor(20./dt);
if max(size(mydata))<Nt;
```

```
    Nt = max(size(mydata));
end
% normalise data:
for m = 1:nCH
    mydata2(m,:) = fBandpass(mydata(m,1:Nt),dt,1,48);
end
mydata = mydata./mean(std(mydata2,[],2),1);
% Compute standard deviation, PLF and phase-lag
for m = 1:nCH
    mySTDband(m) = std(fBandpass(mydata(m,1:Nt),dt,frq(1),frq(2)),[],2);
end;
% compute instantaneous phase and spectral power in each channel:
for m = 1:nCH
    nHil(m,:) = angle(hilbert(fBandpass(mydata(m,1:Nt),dt,frq(1),frq(2))));
    SPower(m) = var(fBandpass(mydata(m,1:Nt),dt,frq(1),frq(2)));
end
% compute PLF and phase-lag:
sr = 256;
for m = 1:nCH
    for n = 1:nCH
        PLF(n,m) = abs(mean(exp(i*(nHil(n,sr:Nt-sr)-nHil(m,sr:Nt-sr)))));
        LAG(n,m) = angle(mean(exp(i*(nHil(n,sr:Nt-sr)-nHil(m,sr:Nt-sr)))));
    end
end
% Compute mean degree biomarker
MeanDeg = mean(sum(PLF));
% Compute Alpha peak
Alpha = fAlphaPeak(mydata,dt);
% Compute local coupling
PLFSparse = fDespur(PLF.*(LAG>0));
phi = LAG;
phi(find(phi>pi/2)) = phi(find(phi>pi/2))-pi;
phi(find(phi<-pi/2)) = phi(find(phi<-pi/2))+pi;
Local = zeros(19,20,1);
for r = 1:20
    myK = 0.4.*r.*mySTDband.^2;
    myK2 = 4;
    for ch = 1:nCH
        Local(ch,r)=abs(mean(fLocal(myK,PLFSparse.*exp(-i.*phi),ch,myK2)));
    end
end
end
% END OF FUNCTION



%%%%%%%%%%%%%%%%%%%%%%%%%%%%%%%%%%%%%%%%%%%%%%%%%%%%%%%%%%%%%%%%%%%%%%%%%%%%%%%
function [signalBp] = fBandpass(signal,dt,lower_freq,upper_freq)
% This function applies a standard bandpass filter to a time series.
% Use: [signalBp] = bandpass(signal,dt,lower_freq,upper_freq)
% Input:
%   signal      - 1D time series
%   dt          - time interval in seconds
%   lower_freq  - lower boundary of band pass
%   upper_freq  - upper boundary of band pass
% Output:
%   signalBp    - bandpass filtered 1D time series
```

```
% maximum frequency of spectrum:
Fs = 1./dt;
% length of time series
N = max(size(signal,1),size(signal,2));
% frequency steps of spectrum:
dF = Fs/N;
% range of frequencies:
f = (-Fs/2:dF:Fs/2-dF)';
% Band-Pass Filter:
BPF = ((lower_freq < abs(f)) & (abs(f) < upper_freq));
% compute FFT of time series:
signal = signal-mean(signal);
spctrm = fftshift(fft(signal));
% apply bandpass filter to FFT of time series:
if size(BPF,1) == size(spctrm,1)
    spctrm = BPF.*spctrm;
else
    spctrm = BPF'.*spctrm;
end
% compute IFFT to obtain bandpass filtered signal:
signalBp = ifft(ifftshift(spctrm));
end
% END OF FUNCTION



%%%%%%%%%%%%%%%%%%%%%%%%%%%%%%%%%%%%%%%%%%%%%%%%%%%%%%%%%%%%%%%%%%%%%%%%%%%%%
function [fPeak] = fAlphaPeak(eegdata,dt)
% This function computes the alpha peak frequency from an EEG recording by
% searching for the maximum of the power spectrum in channels O1 and O2,
% in the frequency range of 8-13Hz. The alpha peak frequencies of channels
% O1 and O2 are averaged.
% AUXILIARY FUNCTION:
%   fSpectrum: computes frequency spectrum of a time series
% prepare data:
if size(eegdata,1)>size(eegdata,2)
    eegdata = eegdata';
end
% Compute spectrum of O1:
[spec1,~] = fSpectrum(eegdata(9,:),dt);
% Compute spectrum of O2:
[spec2,ff] = fSpectrum(eegdata(10,:),dt);
% find alpha frequency range (8-13Hz):
af = find((ff>=8)&(ff<=13));
mf = ff(af);
% find peak frequency in O1:
[~,id1] = max(spec1(af));
peak1 = mf(id1);
% find peak frequency in O2:
[~,id2] = max(spec2(af));
peak2 = mf(id2);
% compute average:
fPeak = (peak1+peak2)./2;
end
% END FUNCTION
```

```
%%%%%%%%%%%%%%%%%%%%%%%%%%%%%%%%%%%%%%%%%%%%%%%%%%%%%%%%%%%%%%%%%%%%%%%%%%%%%%
function [outM] = fDespur(M)
% This function removes spurious connections from the adjancency matrix,
% using the algorithm from Schmidt et al. 2014, PLoS CB.
% This algorithm removes a connection between two nodes a and b, if there
% exist stronger, indirect connections via other nodes. For example, if the
% connections (a-->c) and (c-->b) are larger than (a-->b), then (a-->b) is
% assumed to be spurious (i.e. a result of the connections (a-->c) and
% (c-->b)), and is therefore removed (set to zero).
% This algorithm also removes connections that can be explained by indirect
% connections via two other nodes (i.e. (a-->c), (c-->d), (d-->b)). Whether
% a connection exists or not is indicated by the array 'connmatrix'
% (containing a 1 or a 0).
N = size(M,1);
connmatrix = ones(N,N);
for m = 1:N
    for n = 1:N
        if M(m,n)>0
            for o = 1:N
                if(M(m,o)>M(m,n))&&(M(o,n)>M(m,n))
                    connmatrix(m,n) = 0;
                end
                for p = 1:N
                    if((M(m,o)>M(m,n))&&(M(o,p)>M(m,n)))&&(M(p,n)>M(m,n))
                        connmatrix(m,n) = 0;
                    end
                end
            end
        end
    end
end
outM = M.*connmatrix;
end
% END OF FUNCTION

%%%%%%%%%%%%%%%%%%%%%%%%%%%%%%%%%%%%%%%%%%%%%%%%%%%%%%%%%%%%%%%%%%%%%%%%%%%%%%
function out = fLocal(K,Cmat,Nnum,K2)
% This function computes the order parameters in the modular Kuramoto model
% that we use to determine the Local Coupling biomarker, for a given set of
% parameters and a given channel location. It uses the MATLAB inbuilt
% function 'fsolve' to numerically solve the set of nonlinear equations.
% Input:
%   K     - the vector of local coupling parameters
%   Cmat  - connectivity matrix
%   Nnum  - channel location that is to be put into the synchronous state
%   K2    - value of the local coupling parameter in the selected channel
% Output:
%   out   - vector of order parameters, determined by fsolve
function [out] = fNonLinR(x)
    Km = K;
    Km(Nnum) = K2;
    Cmat2 = diag(Km) + Cmat;
    xTR = transpose(x);
    out = abs(-xTR + 0.5.*Cmat2*xTR - 0.5.*(conj(Cmat2)*xTR).*xTR.^2);
end
options = optimset('Display','off','TolFun',1e-6,'TolX',1e-6);
out = fsolve(@fNonLinR,0.5.*ones(1,size(Cmat,1)),options);
end
```

```
% END OF FUNCTION



%%%%%%%%%%%%%%%%%%%%%%%%%%%%%%%%%%%%%%%%%%%%%%%%%%%%%%%%%%%%%%%%%%%%%%%%%%%
function [SEN,outthr] = fSEN(input1,input2)
% This function computes the maximum specificity at 100% sensitivity.
% Input:
%   input1  - IGE subjects (training set)
%   input2  - controls (training set)
% Output:
%   SEN     - maximum sensitivity
%   outthr  - threshold value at which maximum sensitivity is achieved
% clean input from NaN:
input1 = input1(~isnan(input1));
input2 = input2(~isnan(input2));
% put input into the right format:
if size(input1,2)>size(input1,1)
    input1 = input1';
end
if size(input2,2)>size(input2,1)
    input2 = input2';
end
% compute all possible threshold values:
prot_thresh = sort(cat(1,input1,input2),'ascend');
thresh = (prot_thresh(2:end)+prot_thresh(1:end-1))/2;
thresh = [min(prot_thresh)-0.001; thresh; max(prot_thresh)+0.001];
% compute maximum sensitivity and corresponding threshold value:
if median(input1)>median(input2)
    mthr = min(thresh(find(thresh>max(input2))));
    SEN = sum(input1>mthr(1))./numel(input1);
    outthr = mthr(1);
else
    mthr = max(thresh(find(thresh<min(input2))));
    SEN = sum(input1<mthr(1))./numel(input1);
    outthr = mthr(1);
end
end
% END OF FUNCTION



%%%%%%%%%%%%%%%%%%%%%%%%%%%%%%%%%%%%%%%%%%%%%%%%%%%%%%%%%%%%%%%%%%%%%%%%%%%
function [SPC,outthr] = fSPC(input1,input2)
% This function computes the maximum specificity at 100% sensitivity.
% Input:
%   input1  - IGE subjects (training set)
%   input2  - controls (training set)
% Output:
%   SPC     - maximum specificity
%   outthr  - threshold value at which maximum specificity is achieved
% clean input from NaN:
input1 = input1(~isnan(input1));
input2 = input2(~isnan(input2));
% put input into the right format:
if size(input1,2)>size(input1,1)
    input1 = input1';
end
```

```
if size(input2,2)>size(input2,1)
    input2 = input2';
end
% compute all possible threshold values:
prot_thresh = sort(cat(1,input1,input2),'ascend');
thresh = (prot_thresh(2:end)+prot_thresh(1:end-1))/2;
thresh = [min(prot_thresh)-0.001; thresh; max(prot_thresh)+0.001];
% compute maximum specificity and corresponding threshold value:
if median(input1)>median(input2)
    mthr = max(thresh(find(thresh<min(input1))));
    SPC = sum(input2<mthr(1))./numel(input2);
    outthr = mthr(1);
else
    mthr = min(thresh(find(thresh>max(input1))));
    SPC = sum(input2>mthr(1))./numel(input2);
    outthr = mthr(1);
end
end
% END OF FUNCTION



%%%%%%%%%%%%%%%%%%%%%%%%%%%%%%%%%%%%%%%%%%%%%%%%%%%%%%%%%%%%%%%%%%%%%%%%%%%%%
function [outspec,outf] = fSpectrum(signal,dt)
% This function computes the frequency spectrum of a time series.
% Use: [outspec,outf] = fSpectrum(signal,dt)
% Input:
%   signal  - 1D array representing the signal
%   dt      - time between consecutive time points (inverse sampling rate)
% Output:
%   outspec - spectrum of the signal
%   outf    - frequency space
% maximum frequency:
Fs = 1./dt;
% length of signal:
N = max(size(signal,1),size(signal,2));
% frequency steps:
dF = Fs/N;
% vector of frequencies:
f = (-Fs/2:dF:Fs/2-dF)';
% compute FFT:
signal=signal-mean(signal);
spectron = abs(fftshift(fft(signal)));
% restrict spectrum to non-negative frequencies:
outf = f(find(f>0));
outspec = spectron(find(f>0));
end
% END OF FUNCTION
```
